# P-441. Gut Permeability Response to Inulin Supplementation in Well-Controlled HIV Patients with Metabolic Syndrome

**DOI:** 10.1093/ofid/ofae631.641

**Published:** 2025-01-29

**Authors:** Praveen Kumar vikraman, Ahmed Shamayl, Valli Mani, Rubab Sohail, Meredith Akerman, Cristina Sison, Bruce Hirsch

**Affiliations:** Appalachian Regional Healthcare, Hazard, Kentucky; Northwell - Long Island Jewish Medical Center, New Hyde Park, New York; Donald and Barbara Zucker School of Medicine at Hofstra/Northwell, Forest Hills, New York; Northwell Health, New Hyde Park, New York; Northwell Health, New Hyde Park, New York; Feinstein Institutes for Medical Research, Manhasset, New York; Hoftsa Northwell School of Medicine, Manhasset, NY

## Abstract

**Background:**

HIV infection is known to cause abnormally increased gut permeability resulting in chronic inflammation. Serum levels of Zonulin protein are elevated in persons with increased gut permeability. The objective of this study is to assess whether Inulin (fiber) supplementation will result in a decrease in gut permeability in well-controlled HIV patients with metabolic disorder as measured by baseline and post-intervention Zonulin levels.

Statistical Results

**Figure:**
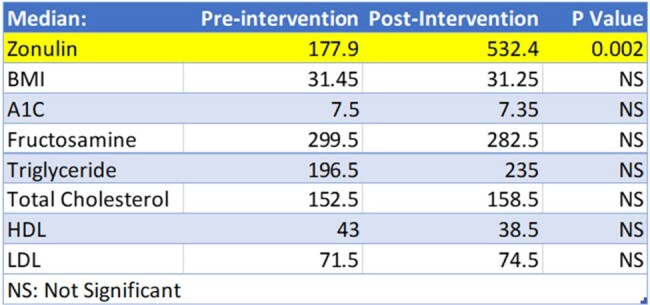

There was a statistically significant increase in median Zonulin levels from baseline to post-intervention (p=0.002)

**Methods:**

10 subjects were selected from patients enrolled in care at the Center for AIDS Research and Treatment (CART) based at North Shore University Hospital. Eligibility criteria included well-controlled HIV patients with evidence of metabolic syndrome. The duration of the study was 6 weeks and the intervention was Inulin supplement use. Bloodwork drawn pre- and post-intervention included Zonulin levels, A1c, Fructosamine, Cholesterol, Triglycerides, HDL, LDL, and non-HDL levels. BMI was also checked. Patients were provided with organic Inulin powder and instructed to take 1 tablespoon once a day for the first week and advance to twice a day for the rest of the study. Every week patients were contacted via telephone to assess compliance and side effects. The primary endpoint was the assessment of baseline and post-intervention Zonulin levels, with the secondary endpoint including baseline and post-intervention BMI, A1c, Fructosamine and Lipids. Descriptive statistics were computed and significance was determined via Wilcoxon signed-rank test.

**Figure:**
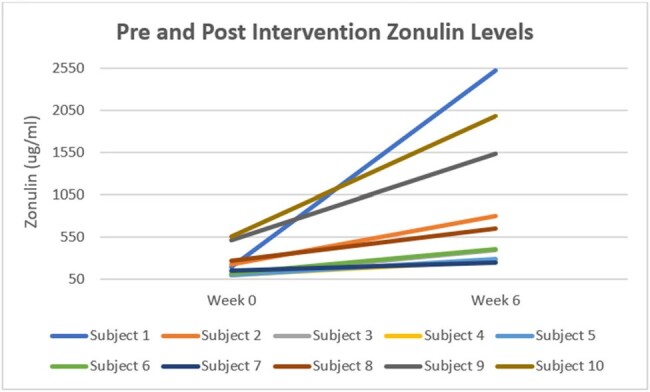

Pre and Post Intervention Zonulin levels in all 10 subjects

**Results:**

Among the patients enrolled 80% were male, 60% were White, 80% were not Hispanic or Latino, and the median age was 60.5. Flatulence and bloating were the most frequent side effects of inulin reported. The median Zonulin levels at baseline were 177.9µg/mL, and post-intervention were 532.4µg/mL. There was a statistically significant increase in Zonulin levels from baseline to post-intervention (p=0.002). There was no significant difference between baseline and post-intervention for any of the other parameters in the dataset.

**Conclusion:**

Inulin supplementation resulted in a statistically significant increase in Zonulin levels, which goes against our hypothesis. Whether this is due to underlying worsening of pre-existing gut dysbiosis or other factors needs further study.

**Disclosures:**

**All Authors**: No reported disclosures

